# The Evaluation, Diagnosis, and Management of Ovarian Cysts, Masses, and Their Complications in Fetuses, Infants, Children, and Adolescents

**DOI:** 10.3390/healthcare13070775

**Published:** 2025-03-31

**Authors:** Marko Bašković, Dubravko Habek, Luca Zaninović, Ivan Milas, Zenon Pogorelić

**Affiliations:** 1 Department of Pediatric Surgery, Children’s Hospital Zagreb, Ulica Vjekoslava Klaića 16, 10000 Zagreb, Croatia; 2 School of Medicine, University of Zagreb, Šalata 3, 10000 Zagreb, Croatia; 3 Croatian Academy of Medical Sciences, Kaptol 15, 10000 Zagreb, Croatia; 4 Scientific Centre of Excellence for Reproductive and Regenerative Medicine, School of Medicine, University of Zagreb, Šalata 3, 10000 Zagreb, Croatia; 5 Department of Obstetrics and Gynecology, Clinical Hospital Merkur, Zajčeva ulica 19, 10000 Zagreb, Croatia; 6 School of Medicine, Catholic University of Croatia, Ilica 242, 10000 Zagreb, Croatia; 7 Department of Obstetrics and Gynecology, University Hospital Centre Zagreb, Petrova ulica 13, 10000 Zagreb, Croatia; 8 Department of Surgical Oncology, University Hospital for Tumors, University Hospital Centre Sestre Milosrdnice, Ilica 197, 10000 Zagreb, Croatia; 9 Department of Pediatric Surgery, University Hospital of Split, Spinčićeva ulica 1, 21000 Split, Croatia; 10 School of Medicine, University of Split, Šoltanska ulica 2a, 21000 Split, Croatia

**Keywords:** ovary, ovarian cyst, ovarian mass, cystectomy, oophorectomy, gynecology, pediatric surgery, fetus, infant, children, adolescent

## Abstract

The majority of abdominal masses in female children derive from the ovaries. Ovarian masses in pediatric populations can vary from simple functional cysts to malignant neoplasms. Their incidence, clinical presentation, and histological distribution vary across age groups. In the assessment of ovarian masses in children, the primary aim is to determine the probability of malignancy, as the treatment approaches for benign and malignant lesions are significantly distinct. The primary imaging tool for evaluating ovarian cysts and masses is ultrasound, which can assess the size, location, and characteristics of masses. Magnetic resonance imaging (MRI) or computed tomography (CT) may be used for further evaluation if ultrasound findings are inconclusive or if malignancy is suspected, especially in older adolescents. Serum markers may be considered in older adolescents to help assess the risk of malignancy, though it is less useful in younger populations due to normal developmental variations. Many functional ovarian cysts, especially those detected in fetuses or infants, often resolve spontaneously without intervention. Surgical intervention is indicated in cases of large cysts that cause symptoms, or if there are concerns for malignancy. Common procedures include primarily ovarian sparing laparoscopy or laparotomy. Complications like torsion, rupture, or hemorrhage may require urgent surgical intervention. Treatment should be performed in specialized centers to avoid unnecessary oophorectomies and ensure the best possible outcome for the patient. This comprehensive review aims to provide an overview of the evaluation, diagnosis, and treatment of ovarian masses in the pediatric population. Emphasis is placed on the particularities of the lesions and their management in relation to age subgroups.

## 1. Introduction

Ovarian lesions, ranging from simple functional cysts to malignant tumors, make up the majority of abdominal masses in the pediatric population [1,2]. Ovarian cysts represent 60% of all ovarian lesions in this population, while true ovarian neoplasms are rare, with an incidence of 2.6 cases per 100,000 girls per year. Only 3–8% of adnexal masses show a malignant character, accounting for 1–2% of all pediatric cancers [3,4,5,6]. Ovarian neoplasms are classified according to their histological origin into three groups—germ cell, sex cord-stromal, and epithelial tumors. Their incidence, etiology, and clinical presentation differ significantly between age groups. In children and adolescents, germ cell tumors are the most common ovarian neoplasms, unlike epithelial tumors in adults. Mature cystic teratomas are the most prevalent tumor type. Dysgerminomas, on the other hand, are the most common malignant ovarian neoplasms in the pediatric population [7]. Functional ovarian cysts show a bimodal distribution, with peaks in the fetal/neonatal and perimenarchal periods, as their formation is influenced by hormonal stimulation, whether by maternal gonadotropins or by the activation of the hypothalamic–pituitary–ovarian axis [8].

Adnexal masses are typically identified through imaging studies, with ultrasound being the modality of choice for initial evaluation. Further diagnostics are usually performed by magnetic resonance imaging, which allows for the excellent visualization of soft tissue organs while avoiding the use of ionizing radiation [3,4]. Imaging features, together with biochemical evaluation (levels of serum tumor markers like alpha-fetoprotein (AFP), lactate dehydrogenase (LDH), beta subunit of human chorionic gonadotropin (β-hCG), cancer antigen 125 (CA-125), inhibin A and B), are crucial in the assessment of the risk of malignancy, as the management of benign and malignant lesions is essentially different [9]. During the surgical treatment of ovarian masses in children, the emphasis should be on the preservation of fertility using minimally invasive laparoscopic surgical techniques and an ovary-sparing approach when feasible [10].

This comprehensive review aims to provide an overview of the evaluation, diagnosis, and treatment of ovarian masses in the pediatric population. Emphasis is placed on the particularities of the lesions and their management in relation to age subgroups.

## 2. Ovarian Cysts

### 2.1. Fetuses

Fetal ovarian cysts, as the most common abdominal cysts in female fetuses with a frequency of 1:2500, are usually diagnosed in the third trimester [11,12]. They typically present as functional or benign cysts, and development is attributed to increased levels of fetal gonadotropins, maternal estrogens, and placental human chorionic gonadotropins [13]. Ovarian cysts < 20 mm in diameter are considered physiological, representing a maturing follicle, while cysts > 20 mm in diameter are considered abnormal and are classified as simple (round, anechoic, unilocular, and thin-walled) or complex (thick-walled with heterogeneous echogenicity, fluid-debris level, and intracystic septations) [12,14]. Differential diagnosis, by way of the careful observation and determination of the organ of origin and the position and appearance of surrounding structures, should distinguish them from simple renal cysts, multicystic dysplastic kidneys, hydronephrosis, urachal cysts, hydrocolpos, enteric duplication cysts, meconium pseudocysts, choledochal cysts, lymphatic malformations, and cystic sacrococcygeal teratoma [15,16]. Magnetic resonance imaging is potentially useful in cases such as maternal obesity, fetal malposition, and oligohydramnios [12]. A characteristic finding of fetal ovarian cysts on ultrasonography is a daughter cyst, which is a small, round, and anechoic structure within the cyst [17], but the diagnosis is usually based on the following ultrasound criteria: female sex, non-midline regular cystic structure, normal-appearing urinary tract, and normal-appearing gastrointestinal tract [11]. The highest priority in perinatal management is to carefully monitor the development of the cyst to avoid potential torsion and subsequent ovarian necrosis and other possible complications, such as cyst compression on other intra-abdominal organs [18,19,20]. The optimal treatment of fetal ovarian cysts remains controversial to date. While decompression of the cyst by percutaneous aspiration has been suggested on the one hand, particularly for cysts of ≥40 mm in diameter or for those with rapid growth, defined as ≥10 mm per week, on the other hand, a conservative expectant approach has been advocated, given the potential for spontaneous resolution, particularly in simple cysts [8,21,22,23,24,25,26]. The presence of a fetal ovarian cyst should not affect the timing and method of delivery because the risk, timing, and duration of torsion cannot be predicted [12,19].

### 2.2. Infants

**Figure 1:**
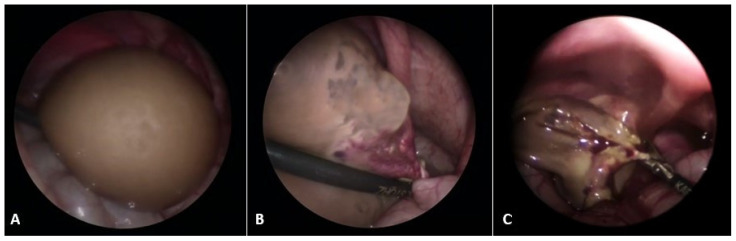
A 7-day-old female neonate presented with a large abdominal mass. (**A**) Laparoscopic examination revealed a necrotic, twisted, huge neonatal ovarian cyst measuring 6 × 5 × 4 cm; (**B**) Twisting of the ovarian pedicle by 720 degrees; (**C**) After decompression of the cyst, a laparoscopic salpingo-oophorectomy was performed due to the obvious necrosis of the ovarian tissue. Source: Archive of the Department of Pediatric Surgery, University Hospital of Split.

The spontaneous regression of ovarian cysts usually occurs after birth, usually by 6 months or even 1 year of age [26,27]. The rate of spontaneous regression tends to decrease with increasing cyst size. The rate ranges from approximately 90% for cysts ≤ 2.9 cm to 20% for those ≥ 6 cm [28,29]. Accordingly, ultrasound observation after the child’s birth is recommended every 4–6 weeks. For cysts that enlarge or persist for 4 months or more, continued observation, aspiration, or surgical removal (cystectomy) are all reasonable choices [30]. Aspiration is a possible choice for uncomplicated neonatal ovarian cysts that are ≥ 6 cm in diameter, provided they have not spontaneously regressed by four months. Should the cyst recur, it can be reaspirated or surgically removed (usually for neonatal ovarian cysts that are complex, symptomatic, or enlarging). Laparoscopic surgery is a recommended surgical approach, wherein ovarian tissue must be preserved as much as possible [11,30,31,32,33]. Ovarian torsion is the most common complication of ovarian cysts in infants (Figure 1), and it occurs mainly in cysts that begin to grow rapidly and their ultrasound features change. Also, the risk of ovarian torsion has been found to be more related to the length of the pedicle than to the size of the cyst itself [30,34,35].

### 2.3. Children

In the prepubertal period, the incidence of ovarian cysts is low unless there is a disease affecting ovarian cysts, since ovarian stimulation by gonadotropins reduces after the neonatal period and typically stays low until puberty [36]. Factors leading to ovarian cysts in prepubertal girls encompass patients with idiopathic central precocious puberty, McCune Albright syndrome, thyroid disorders, and hormonally active ovarian tumors. However, the majority of simple ovarian cysts in children are related to the failure of an ovarian follicle to regress [37,38,39]. In prepubertal children, ovarian cysts typically appear as an asymptomatic abdominal mass causing abdominal distension or are revealed incidentally during radiographic assessments. If symptoms are present due to a larger cyst, they usually present as chronic periumbilical or lower quadrant pain and may also present with bloating, urinary frequency or retention, constipation, or a feeling of abdominal fullness. Acute severe pain is usually due to torsion, perforation, hemorrhage, or infarction [36]. In prepubertal girls, ovarian cysts should be distinguished by ultrasound from paraovarian, mesothelial, and broad ligament cysts [40,41]. The management of ovarian cysts in prepubertal girls is based on the ultrasound appearance of the cyst and associated clinical findings. For asymptomatic patients, observation is the method of choice, but children and their parents should be counseled about the signs and symptoms of ovarian torsion so that they can seek emergency care without delay. In observational studies in the prepubertal period, cysts up to 9 cm in diameter usually resolve spontaneously, and indications for surgical exploration include ovarian cysts ≥ 9 cm in diameter, which are at increased risk of malignancy, ultrasound features of a tumor process (e.g., septation, increased solid tissue, calcification), ovarian torsion, and acute rupture with bleeding and hemodynamic instability [42,43,44,45].

### 2.4. Adolescents

**Figure 2:**
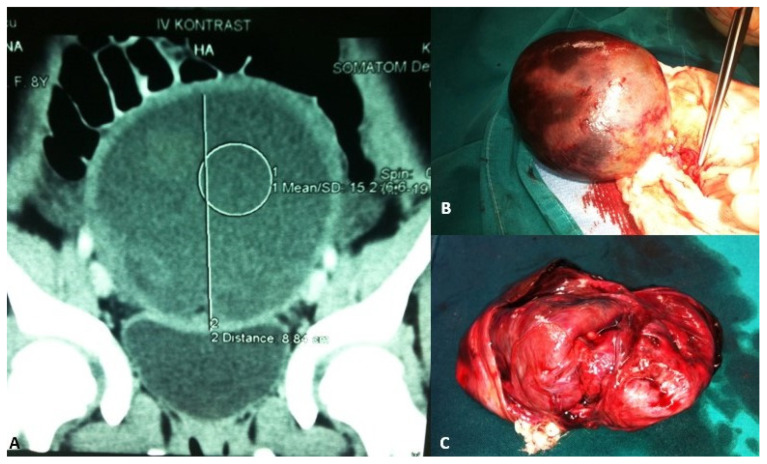
A 13-year-old adolescent presented to the emergency room because of recurrent crampy abdominal pain that lasted a short time and then subsided. She had had the symptoms for about a week. An abdominal ultrasound was performed, which showed a mass in the pelvis. (**A**) MSCT of the abdomen revealed a 9.1 × 8.8 cm solid cystic mass in the right ovary that was not perfused; (**B**) Intraoperatively, a torquing, partially necrotic tumor of the ovary was noted; (**C**) Pathohistological findings were consistent with a mature teratoma of the right ovary. Source: Archive of the Department of Pediatric Surgery, University Hospital of Split.

Adolescent ovaries can have several follicles at various developmental stages. The majority of ovarian cysts in adolescents are follicular cysts, which occur due to a maturing follicle’s failure to ovulate and involute. Large cysts can lead to frequent urination, constipation, or a sensation of pressure in the lower belly, whereas twisting, rupture, or hemorrhage of the cyst causes intense acute pain (Figure 2) [46].

**Figure 3:**
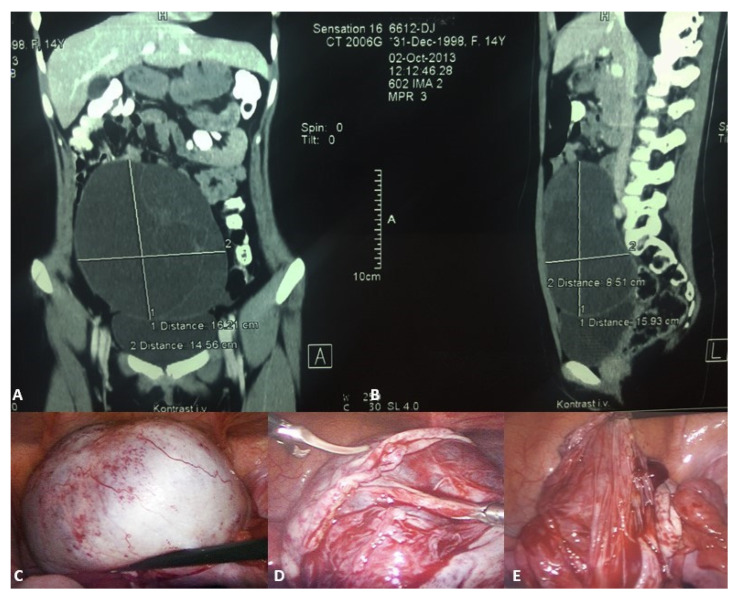
A 14-year-old girl presented with intermittent abdominal pain, loss of appetite, and a palpable abdominal mass. (**A**) Multislice computed tomography revealed a giant right abdominal ovarian cyst measuring 16.2 × 14.6 cm; (**B**) The cystic mass filled the entire lower abdomen and was pressing on the surrounding structures; (**C**) Laparoscopic examination revealed a giant ovarian cyst; (**D**) The ovarian cortex was opened, 2 L of clear contents were aspirated; (**E**) A laparoscopic cystectomy with ovarian sparing was performed. A pathohistological examination revealed a simple ovarian cyst. Source: Archive of the Department of Pediatric Surgery, University Hospital of Split.

In the phase of adolescence, the possibility of obstructive genital anomalies, ovarian tumors, tubal conditions, uterine masses, and urologic and gastrointestinal conditions should be considered in the differential diagnosis. Ultrasound is the initial imaging modality in differentiating ovarian cysts from other possible conditions [2,47]. As for follicular cysts in adolescent girls, they usually disappear spontaneously within eight weeks. At this age, monophasic combination estrogen/progestin oral contraceptive pills (OCPs) containing ≥35 mcg ethinyl estradiol are commonly recommended, as they inhibit the ovarian-hypothalamic axis and can stop ovulation and the development of a new functional cyst [48,49]. Indications for laparoscopic cystectomy or aspiration are persistence for ≥3 months, size ≥ 6 cm (simple cysts measuring 6 to 12 cm may resolve spontaneously and can be monitored safely in certain patients), pelvic pain, or urinary frequency. Because of the high recurrence rate in the case of aspiration, laparoscopic cystectomy is usually preferred [50,51,52]. For corpus luteum cysts, observation for three months is also recommended for asymptomatic patients. Monophasic combination estrogen/progestin OCPs with ≥35 mcg ethinyl estradiol are also prescribed. Cystectomy alone is rarely warranted. Persistent or non-involuting ovarian cysts should be treated through cystectomy and ovarian tissue preservation (Figure 3) [49,53,54].

## 3. Ovarian Masses

Even though the majority of ovarian masses in children are either functional ovarian cysts or benign ovarian tumors, early diagnosis impacts treatment outcomes for children with malignant tumors. Ovarian masses are usually presented as an incidental radiological finding, or depending on the age and size and pathology of the mass itself, they may present with abdominal pain, increased abdominal girth, a palpable mass in the abdomen or pelvis, symptoms related to compression of other organs (e.g., nausea, vomiting, abdominal fullness, constipation, lower abdominal pressure, urinary frequency or retention), precocious puberty (virilization), menstrual irregularities, and paraneoplastic or autoimmune syndrome [55,56,57,58]. Ovarian tumors, whether benign or malignant, are rare in children and adolescents, representing merely 1–2% of all tumors, with an incidence of roughly 2.6 per 100,000 girls annually [2]. Although malignant ovarian tumors are rare in children and adolescents, around 10–20% of surgically treated ovarian tumors were found to be malignant [14,59,60,61,62]. Clinical characteristics more frequently linked to malignant tumors rather than benign ones include bilateral masses, fixed masses with irregular borders, ascites, and complaints of precocious puberty [63].

### 3.1. Germ Cell Tumors

**Figure 4:**
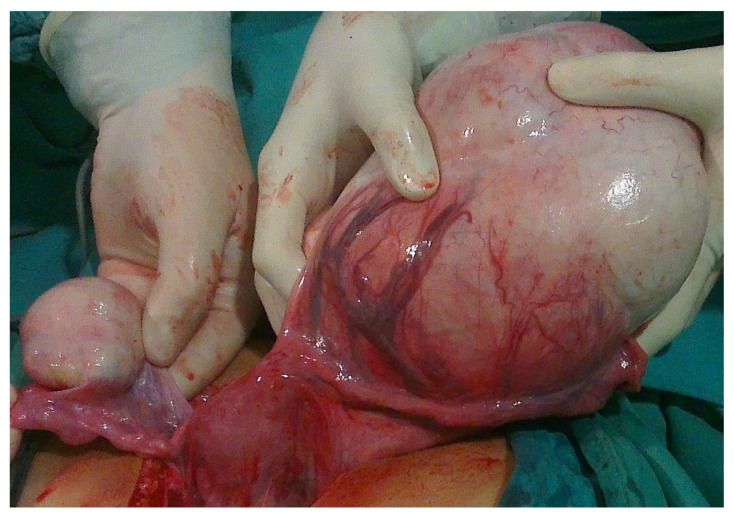
An 11-year-old girl noticed that her stomach was growing along with pain, nausea, and a palpable mass. Ultrasound and MRI revealed bilateral ovarian tumor inhomogeneous solid cystic masses similar to dermoid, left 13 cm, right 5 cm in diameter. Tumor markers were not increased, and due to the size of the tumor, a laparotomy approach was decided. Intraoperative findings: bilateral asymmetrical ovarian masses and bilateral cystectomy were performed. A pathohistological examination revealed a mature teratoma. Source: Archive of the Department of Obstetrics and Gynecology, Clinical Hospital Merkur.

**Figure 5:**
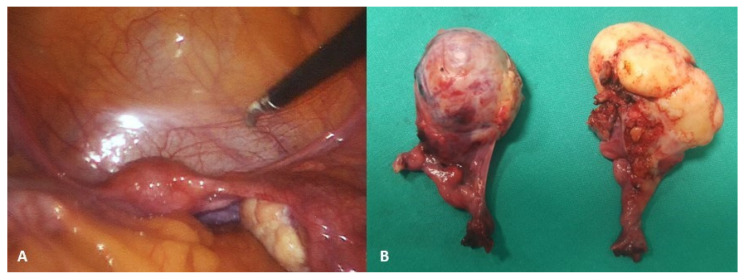
A 17-year-old female patient presented to a pediatric endocrinologist for primary amenorrhea. After diagnostic evaluation, the endocrinologist diagnosed a female phenotype and a male karyotype (46, XY). The MR scan of the abdomen showed a tumor in both gonads. The left gonad looked like a testis and the right one like an ovary. A diagnosis of a disorder of sexual development (DSD) was made. A bilateral laparoscopic adnexectomy was performed. The pathohistological examination revealed a bilateral gonadoblastoma with components of a dysgerminoma. No gonadal tissue was present in either of the removed specimens. TNM: T1bNXMX; Figo classification: 1b; (**A**) Intraoperative findings; (**B**) Macroscopic specimen after bilateral adnexectomy. Source: Archive of the Department of Pediatric Surgery, University Hospital of Split.

Most ovarian tumors (35–45%) in children and adolescents are derived from germ cells (e.g., mature teratomas (Figure 4), immature teratomas, yolk sac tumors (YSTs), gonadoblastomas, dysgerminomas (Figure 5), embryonal carcinoma, non-gestational choriocarcinoma, and mixed germ cell tumors), of which 80% of germ cell tumors (GCTs) are benign, while only 20% are malignant. They arise due to variations from the normal differentiation of germ cells [59,64]. Mature and immature teratomas account for approximately 80% of all ovarian GCTs and are bilateral in 5% of cases. The most common malignant ovarian GCTs in children are YSTs, while dysgerminomas are the most common malignant entity in adolescence, with bilaterality in 10% of cases. Gonadoblastoma is a rare GCT seen in girls with dysgenetic gonads [65,66].

### 3.2. Epithelial Tumors

**Figure 6:**
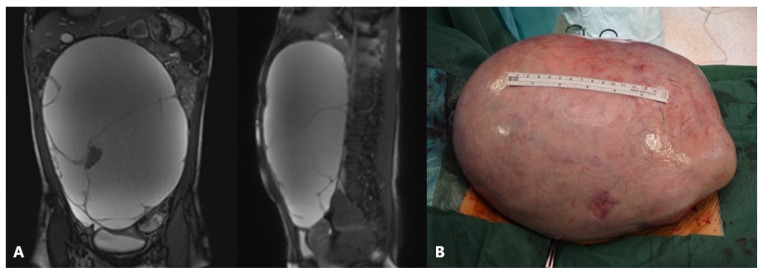
A 14-year-old girl presented with abdominal distension that had been present for several months. Apart from occasional constipation, she had no other symptoms. (**A**) MRI of the abdomen shows an intraperitoneal mass inseparable from the right ovary, compressing the pancreas and abdominal aorta; (**B**) Surgical exploration of the abdomen—mature cystic ovarian teratoma with foci of adenocarcinomas measuring 28 × 22 × 14 cm. Source: Archive of the Department of Pediatric Surgery, Children’s Hospital Zagreb.

Epithelial tumors (e.g., serous or mucinous cystadenoma), with a frequency of less than 20% in childhood, are rare in prepubertal children. Each of these tumor types may be characterized as benign, malignant, or of low malignant potential [67]. Bilateral involvement is present in up to 20% of cases, and malignant tumors, such as cystadenocarcinoma or undifferentiated carcinoma, are very rare and usually have an aggressive clinical behavior (Figure 6). Borderline ovarian tumors have been described and also labeled as tumors with low malignant potential [68,69,70].

### 3.3. Sex Cord-Stromal Tumors

Sex cord-stromal tumors (e.g., thecomas, fibromas, juvenile granulosa cell tumor, Sertoli–Leydig cell tumors) are rare in children and adolescents, with approximately 5% of ovarian malignancies [71]. Unlike epithelial and germ cell tumors, they often appear with signs of hormone production, such as hirsutism and virilization, menstrual changes, or precocious puberty. A subset of sex cord-stromal tumors is associated with genetic aberrations, and an underlying cancer predisposition should always be considered [72,73].

### 3.4. Imaging

Transabdominal ultrasound serves as the primary tool for evaluating adnexal masses in children and adolescents, allowing for the assessment of the mass’s size and origin (e.g., ovarian, paraovarian), consistency (e.g., cystic, solid), laterality, and any related findings (e.g., ascites, lymphadenopathy), while blood flow can be assessed by Doppler ultrasound [74]. The radiologist should utilize different sonographic methods and integrate the patient’s pertinent medical history and clinical information to assist in identifying the mass and differentiating between normal physiology (a normal component of ovulation) and a pathological condition. Meta-analyses of observational studies indicate that the simultaneous evaluation of ovarian masses using gray-scale morphology alongside color Doppler assessment is more effective than using either morphologic assessment, spectral Doppler assessment, or color Doppler assessment on its own [75,76]. The aim of ultrasound is not to establish if the mass is “definitely” benign or malignant but instead to assess whether the mass is “almost certainly benign” or if it carries a “reasonable chance of being malignant”. There is no universally accepted classification system for adnexal masses. The two systems are the International Ovarian Tumor Analysis (IOTA) Simple Rules and the Ovarian-Adnexal Reporting and Data System (O-RADS) for ultrasound classification [77,78].

In an ultrasound evaluation, simple cysts, which are usually physiological, appear anechoic, lacking septations, solid parts, or mural nodules. They may present with ≤1 peripheral calcification and without Doppler flow. Mucinous and serous cystadenomas are a common cause of simple ovarian cysts in children and adolescents. Complex ovarian masses are cystic and display solid nodular or papillary characteristics (<50 percent), thickened walls, septations (>2 to 3 mm), multiple calcifications, or mural nodules. Factors that may cause sudden complex ovarian masses include ovarian torsion, tubo-ovarian abscess, and ectopic pregnancy, while reasons for persistent complex ovarian masses include mature teratomas, immature teratomas, and endometriomas. Masses that are primarily solid (i.e., ≥50 percent solid components) are deemed malignant until histological evaluation indicates otherwise. In children and adolescents, solid ovarian masses can arise from germ cell tumors (e.g., dysgerminoma) and sex cord-stromal tumors (e.g., juvenile granulosa cell tumors, Sertoli–Leydig cell tumors) [62,67,79,80,81]. Ultrasonographic characteristics that are more indicative of malignant tumors encompass size ≥ 8 to 10 cm, multiple lesions, bilateral masses, solid or heterogeneous (solid components > 2 cm, thick septations, papillary projections), invasive or metastatic, presence of calcifications, ascites, and increased blood flow [60,63,82,83]. If the origin of the mass remains unclear post-ultrasound, or if the tumor is sizable or thought to be malignant, further details (e.g., pelvic lymph nodes, lung or liver metastases) can be acquired through computed tomography (CT) or magnetic resonance imaging (MRI) [84,85].

### 3.5. Laboratory Findings

Helpful markers for studying ovarian tumors in children and adolescents include AFP for immature teratomas, yolk sac tumors, embryonal carcinoma, and Sertoli–Leydig cell tumors (rare); LDH for dysgerminomas; β-hCG for dysgerminomas (rare), embryonal carcinoma, and non-gestational choriocarcinoma; CA-125 for malignant epithelial tumors; and inhibin A and B for juvenile granulosa cell tumors [2]. Estradiol and testosterone are measured to assess hormonally active tumors (e.g., in individuals with early puberty or signs of virilization) [86]. The use of an ovarian tumor marker panel increases sensitivity and specificity. However, the absence of elevated tumor markers does not exclude malignancy, and elevated tumor markers may be present in benign tumors. As a result, determining tumor markers cannot definitively confirm or rule out malignancy. In the context of pediatric ovarian tumors, the usefulness of human epididymis protein 4 (HE4) is still being explored and is not as well established as in adults, and understanding its role could enhance the understanding of tumor biology in children [87,88]. Similarly, carcinoembryonic antigen (CEA) is more commonly associated with adult cancers, while its role in pediatric malignancies, including ovarian tumors, is less well-defined. Values can vary considerably depending on tumor type, age, and other individual factors [89]. The ROMA (Risk of Ovarian Malignancy Algorithm) index is a valuable tool used in the evaluation of ovarian tumors. Its application in the pediatric population has limitations primarily due to the different biological behavior of ovarian tumors in this age group. More research is needed to determine its diagnostic accuracy and clinical utility [90]. When assessing the risk of malignancy in pediatric patients with ovarian tumors, a multifactorial approach that considers clinical presentation, imaging and laboratory findings, and histology, alongside emerging scoring systems, is essential. It is worth mentioning the Gynecologic Oncology Group (GOG) Staging System, which is used to stage ovarian tumors in children, providing a framework for determining the extent of disease and guiding treatment. Furthermore, the Children’s Oncology Group (COG) developed protocols specifically for pediatric tumors, including ovarian tumors. They provide risk stratification based on factors such as age, tumor type, stage at diagnosis, and histological features. Although primarily used in adults, the International Federation of Gynecology and Obstetrics (FIGO) Staging System also applies in pediatric populations and helps to classify the stages of ovarian tumors based on tumor size and spread [91,92,93]. A definitive diagnosis of an ovarian mass can only be confirmed following its surgical removal and histopathological analysis. The monitoring of serum tumor markers is beneficial for postoperative follow-up since it can indicate potential disease relapse [94,95]. Additionally, the cytology of ascites fluid and the assessment of platelet levels could be beneficial. Increased platelet levels serve as a nonspecific marker of ovarian mass and can assist in the acute evaluation of a twisted ovarian mass (platelet counts typically remain normal in nonmalignant ovarian torsion) [96]. A childhood-detected ovarian neoplasm can be the initial indication of a cancer predisposition syndrome, making the diagnosis crucial since it provides the possibility for genetic counseling and surveillance for the patient and her relatives [97]. Ovarian neoplasms detected in childhood, although rare, can be associated with various genetic conditions such as Turner syndrome, Gorlin syndrome, Li–Fraumeni syndrome, Peutz–Jeghers syndrome, familial breast and ovarian cancer syndrome, etc. [98,99,100].

### 3.6. Management

**Figure 7:**
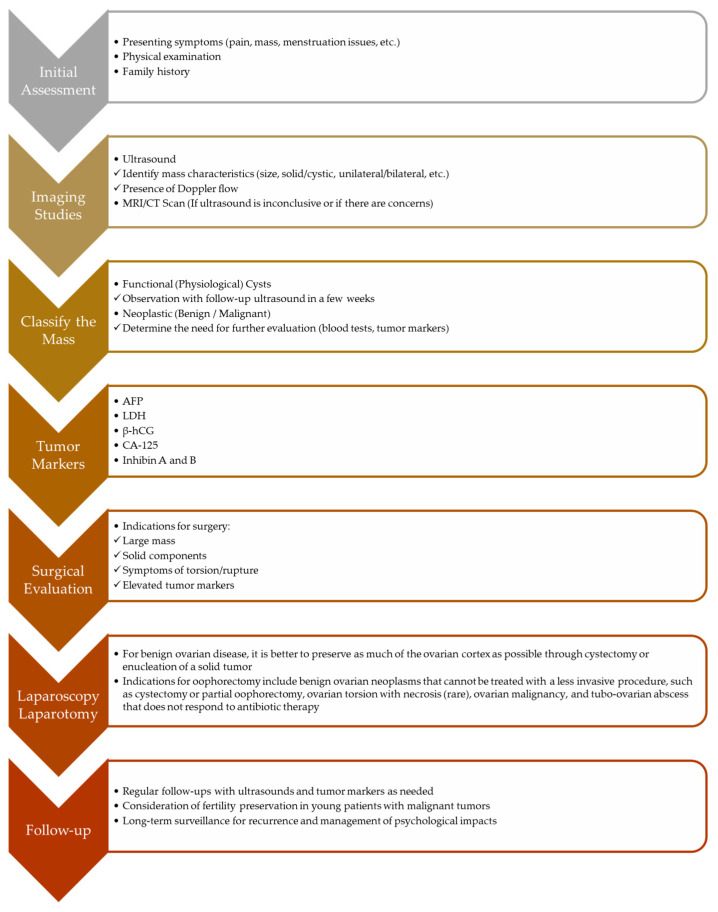
Flowchart for management of pediatric ovarian masses.

The objectives of surgical intervention consist of confirming a definitive diagnosis, complete removal of neoplastic tissue, determining the stage of malignancy, preserving ovarian tissue and function (if possible), and alleviating symptoms (Figure 7) [101]. Maintaining gonadal function is crucial not just for fertility preservation but also for the normal advancement of puberty. Consequently, a conservative surgical approach is utilized to preserve ovarian tissue, except when there is strong suspicion of malignancy (indicated by imaging and elevated tumor markers) or if malignancy is definitively confirmed on a frozen section during the surgery [102,103]. Because abdominal scars can cause psychological problems and reduce self-esteem in young girls, the treatment of ovarian masses in children and adolescents should be minimally invasive, which can be accomplished through ovarian-sparing laparoscopic surgery [104]. Although ovary-preserving surgery is recommended for managing benign ovarian tumors in children and adolescents, 46–54% of these lesions are treated with oophorectomy. Patients treated by pediatric and adolescent gynecologists are less likely to receive unnecessary oophorectomy [61,105]. If malignancy is suspected or confirmed, proper staging includes abdominal and pelvic exploration, peritoneal lavage, biopsies of suspicious areas, and the sampling of pelvic and periaortic lymph nodes [106,107,108].

### 3.7. Differential Diagnosis

The differential diagnosis of adnexal masses in young females is extensive. The differential diagnosis of an intra-abdominal mass in fetuses or neonates includes genitourinary tract disorders (e.g., reproductive tract anomalies, urinary tract obstruction, urachal cysts), gastrointestinal tract disorders (e.g., mesenteric or omental cyst, volvulus, colonic atresia, intestinal duplication), or miscellaneous disorders (e.g., choledochal, splenic, or pancreatic cysts, lymphangioma) [15]. In children and adolescents, the differential diagnosis includes tubal and paratubal cysts, Müllerian abnormalities, tubo-ovarian abscesses, disorders with infectious etiologies (e.g., pelvic inflammatory disease), endometrioma, hydrosalpinx, pyosalpinx, and pregnancy-related masses (ectopic pregnancy). Among the nongynecologic ones, peritoneal inclusion cysts and appendicitis or appendiceal abscess should be singled out (Table 1). Diagnostic algorithms can guide further therapeutic procedures, and signs of an acute abdomen (due to bleeding, torsion, inflammation, or perforation) will indicate urgent surgery, primarily laparoscopic [109,110,111,112].

### 3.8. Follow-Up

Long-term follow-up after surgery for pediatric ovarian tumors is a critical component of post-operative care, focusing on monitoring for recurrence, managing complications, and addressing psychological impacts, particularly in adolescents. Follow-up visits are generally arranged every 3 to 6 months for the first 2 to 3 years post-surgery and then annually for up to 5 years or longer [113,114]. Alongside routine physical examinations, specific tumor markers are monitored, and ultrasound (CT or MRI if necessary) may be included as needed if there are worrisome symptoms or abnormal findings [115]. Postoperative complications can vary depending on the type of tumor and the initial surgical approach (laparoscopy vs. laparotomy). In addition to classic surgical complications and the formation of adhesions, attention should be directed to a possible hormonal imbalance and, consequently, fertility [116,117,118]. The diagnosis and surgery itself can lead to anxiety and depression, especially in adolescent girls, whose self-esteem can be significantly shaken. Psychosocial support from mental health professionals is of utmost importance, both in the short term and in the long term, throughout the recovery process [119,120].

## 4. Complications

### 4.1. Ovarian Torsion

**Figure 8:**
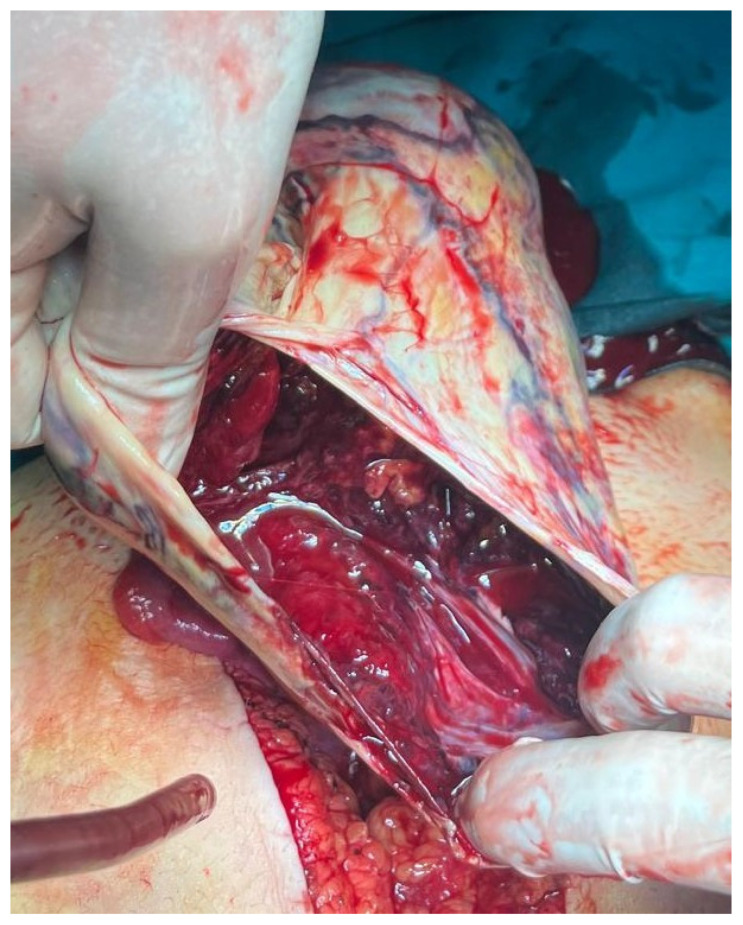
A 14-year-old girl presented with acute abdominal pain and vomiting. Abdominal ultrasonography revealed a left ovarian cystic-solid mass 11 × 8 cm with destruction and free fluid in the abdomen. Because of acute abdomen, exploratory laparotomy and left-side adnexectomy were performed. A ruptured torquated ovarian tumor was found. A pathohistological examination revealed a ruptured ovarian cystic mature teratoma. Source: Archive of the Department of Obstetrics and Gynecology, Clinical Hospital Merkur.

Ovarian torsion occurs in girls of all ages with an incidence of 4.9 per 100,000. It is usually associated with adnexal pathology but can also occur without adnexal pathology (e.g., due to an elongated utero-ovarian ligament), especially in prepubertal girls. Ovarian masses associated with torsion are usually benign (Figure 8) [121]. If torsion happens in utero, the ovary can experience necrosis and transform into a calcified mass or a sessile mass or disappear entirely [46,122]. It commonly manifests in girls as intense one-sided pain in the lower abdomen with a sudden start (or significant irritability with an acute onset in a newborn or young infant), nausea, vomiting, and pallor. In cases of intermittent pain, it may be torsion without complete occlusion of the vascular blood supply [123,124]. There is no specific duration of time after symptom onset that is predictive of certain ovarian necrosis [125]. The timeframe for ovary necrosis to happen varies, but generally, the risk of significant ovarian damage increases as time progresses after the onset of torsion. In summary, while there is individual variability, it is critical to diagnose and treat ovarian torsion as quickly as possible to maximize the chances of ovarian preservation and prevent necrosis [126,127]. The method of choice for the imaging evaluation of ovarian torsion is ultrasound, with the addition of Doppler. Normal Doppler flow does not rule out torsion, as maintained flow may be due to partial occlusion, sporadic intermittent torsion, and collateral circulation (such as utero-ovarian vessels and infundibulopelvic vessels) [128]. The most sensitive sonographic findings are ovarian edema, abnormal ovarian blood flow, whirlpool sign, and relative enlargement of the ipsilateral ovary [129,130]. Computed tomography and magnetic resonance imaging are not routinely performed in the evaluation of ovarian torsion but may be helpful if sonographic findings are equivocal [131,132,133]. The differential diagnosis of ovarian torsion includes conditions such as ectopic pregnancy, ruptured ovarian cyst, tubo-ovarian abscess, and appendicitis [123]. The definitive diagnosis of ovarian torsion is made by direct visualization of the rotated ovary during surgery. Ovarian torsion is a surgical emergency in which most ovaries can be salvaged by untwisting the vascular pedicle. The gold standard is the laparoscopic approach (detorquation, cystectomy with ovarian preservation). In rare cases, oophorectomy is necessary because of severe extensive necrosis or a nonviable gelatinous appearance [134,135]. It is important to note that many patients (even those with blue or black ovaries) retain ovarian function after detorsion [136,137,138]. Therefore, the trend toward ovarian preservation instead of oophorectomy has become accepted as the most appropriate initial treatment for pediatric ovarian torsion. Oophoropexy has been proposed as a means of decreasing future reproductive harm by decreasing the risk of recurrent ovarian torsion, especially in the group of high-risk patients who have had a recurrence of ovarian torsion, those with bilateral ovarian torsion, or idiopathic torsion [139]. The oophoropexy options are a plication of the utero-ovarian ligament (non-fixation technique) and oophoropexy of the ovary to the uterosacral ligament or pelvic sidewall (fixation technique) [140,141].

### 4.2. Fallopian Tube Torsion

Fallopian tube torsion refers to the solitary twisting of the fallopian tube about its axis, occurring without torsion of the ovary. It may happen either in the middle section of the tube itself or around the supportive ligaments of the tube [142]. The clinical presentation can involve generalized or nonspecific pain, either acute or chronic, and is frequently accompanied by nonspecific symptoms like nausea and vomiting. Unrecognized tubal torsion can lead to loss of tubal function and potentially cause hydrosalpinx or necrosis, ultimately resulting in the resorption of the affected tissue [143]. Potential risk factors for tubal torsion include tubal pathology (e.g., hydrosalpinx, segmental tubal agenesis with resulting proximal hydrosalpinx, broad ligament cysts, paratubal cysts, neoplasms, ectopic pregnancy, and congenital anomalies), ovarian mass, infection, altered tubal function (e.g., abnormal peristalsis, spasm), or extrinsic lesions (e.g., adhesions) [144,145]. Radiographically, it can present as an enlarged tubular structure, normal adjacent ovary, or cystic mass separate from the ovary. The diagnosis is typically determined during surgical exploration. Similar to ovarian torsion, performing surgical detorsion can avert permanent ischemic harm and the consequent removal of the fallopian tube [142,146].

### 4.3. Paratubal or Paraovarian Cyst Torsion

**Figure 9:**
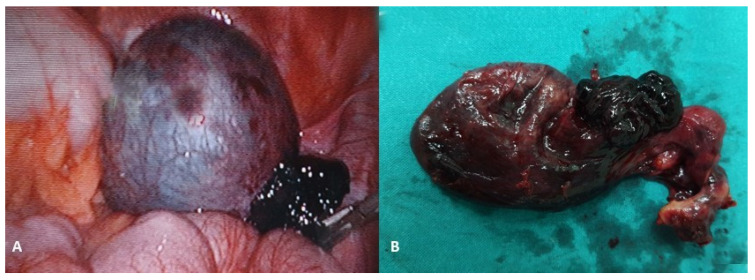
A 15-year-old girl presented with abdominal pain, fever and vomiting. The symptoms started five days prior to surgery. Abdominal ultrasonography revealed a huge cystic mass in the pelvis measuring 13 × 10 × 8 cm adjacent to the left ovary, which was edematous and without visible blood flow. (**A**) A laparoscopic examination revealed a huge paraovarian cyst and torsion of the left adnexa; (**B**) A laparoscopic salpingo-oophorectomy was performed due to the obvious gangrene of the left ovary and fallopian tube. A pathohistological examination revealed a necrotic paraovarian cyst and gangrene of the left ovary. Source: Archive of the Department of Pediatric Surgery, University Hospital of Split.

The torsion of a paraovarian cyst is rare in children. The preoperative diagnosis of a paraovarian cyst is difficult; however, a paraovarian cyst should be suspected if an ipsilateral normal ovary is imaged and the cyst is located next to the uterus. The presence of adnexa torsion in children and adolescents with paraovarian cysts depends on the size of the cysts (Figure 9) [40]. If torsion occurs, the cyst must be carefully excised so as not to endanger the function of the fallopian tubes and ovaries (fertility-sparing surgery). Laparoscopic cyst removal has proven to be an effective surgical approach with favorable outcomes [147,148,149].

### 4.4. Ruptured Hemorrhagic Ovarian Cyst

Rupture of a hemorrhagic ovarian cyst is usually accompanied by acute onset of focal unilateral lower abdominal pain, usually mid-menstrual, and may be accompanied by intraperitoneal bleeding. In cases of massive hemorrhage, shoulder pain or upper abdominal pain is present due to subphrenic blood extravasation [150]. Bleeding can either resolve on its own or be associated with hemodynamic instability. Patients with self-limiting bleeding should be monitored with serial hemoglobin/hematocrit measurements, whereas hemodynamically unstable individuals should be stabilized before surgery, which is frequently performed via laparoscopy. Laparotomy is recommended solely when the surgeon lacks experience in laparoscopic techniques in children or when the patient is hemodynamically unstable and cannot be stabilized [151,152].

## 5. Cystectomy

When surgery is necessary for benign ovarian disease, it is generally better to preserve as much of the ovarian cortex as possible through cystectomy or enucleation of a solid tumor rather than performing an oophorectomy. Aspiration of the cyst contents alone is not recommended because it does not provide tissue for histopathology, and cytology of the cyst fluid is not reliable in ruling out malignancy. When the ovary cannot be preserved or after attempts at conservation yield inadequate viable tissue, an oophorectomy is usually performed [117,153,154].

In open cystectomy, an elliptical incision is created through the delicate ovarian cortex along the ovary’s axis and its most dependent area, steering clear of the fallopian tube and the fimbria ovarica [155]. It is essential to separate the cyst wall from the ovarian cortex. If the cyst’s base is extremely near the ovarian stroma, it is essential to remove it using scissors or electrocautery. It is better to extract the cyst as a whole. If the cyst bursts during handling, it is essential to manage the leakage to prevent contamination of the abdominal cavity [156]. Additionally, once the cyst bursts, the abdominal cavity needs to be thoroughly cleaned to reduce the chances of complications like pseudomyxoma peritonei and chemical peritonitis [157]. After cystectomy, the ovarian tissues can be left open, which is preferable, or they can be reapproximated if hemostasis is required, avoiding sutures on the ovarian capsule. If multiple cysts are present, an attempt should be made to make the fewest possible incisions on the surface of the ovary [12].

In laparoscopic cystectomy, the utero-ovarian ligament is grasped, and the ovary is stabilized. Intact removal of the cyst is preferred if the cyst can be dissected from the surrounding ovarian cortex. Blunt dissection, or occasionally electrocautery, is usually attempted until the cyst is completely removed. If difficulties arise, a cystotomy may be performed, during which it is necessary to control for possible leakage of the cyst contents. The cyst contents should be aspirated completely, and the cyst cavity should be thoroughly rinsed. The cyst wall should then be examined in detail, and any suspicious areas should be attempted to be separated by blunt or sharp dissection. Areas that cannot be separated can be fulgurated. To reduce the risk of intraperitoneal spillage, laparoscopically guided minilaparotomy can be used for large cysts [52,151,158,159,160].

## 6. Oophorectomy

Indications for oophorectomy include benign ovarian neoplasms that cannot be treated with a less invasive procedure such as cystectomy or partial oophorectomy, ovarian torsion with necrosis (rare), ovarian malignancy, and tubo-ovarian abscess that does not respond to antibiotic therapy [161]. Minimally invasive surgery (MIS) is the preferred method for oophorectomy due to its association with reduced complications, quicker recovery, and lower expenses compared to laparotomy. Circumstances where laparotomy might be preferable over MIS involve dense adnexal adhesions, significantly enlarged ovary, or a strong suspicion of malignancy [162,163,164,165,166]. Emerging minimally invasive techniques, including robot-assisted laparoscopy and single-port laparoscopy, are still being refined, particularly for the pediatric population [167,168,169]. No matter the approach used, once access to the abdomen is gained, pelvic and abdominal lavages are collected and stored for staging if malignancy is later confirmed. Additionally, during the abdominal examination, any questionable lesion must be biopsied and dispatched for frozen section analysis. Through dissection, the broad ligament is opened, allowing the ureter to be identified on its medial or posterior side. If the fallopian tube is to be removed, the ovarian vessels are then lifted to enable clear visibility of the nearby ureter. The vessels are clamped and separated, and the pedicles are tied 1 to 2 cm above the ovary to achieve hemostasis and total removal of the ovary. To preserve the fallopian tube, the various tubal branches of the ovarian artery must be systematically clamped and ligated below and parallel to the tube during an open procedure. When done laparoscopically, the pedicles are either sealed or coagulated and then cut [170,171]. Ureteral injuries have been documented during open or minimally invasive surgical oophorectomy when the ureter was not properly identified. Although the ureter is visible through the peritoneum in many patients, there are instances where it is essential to open the peritoneum and conduct a different dissection to expose it [172,173]. Following oophorectomy, patients with a single ovary, in contrast to those with two, exhibited a reduced quantity (e.g., fewer follicles) but comparable quality (e.g., pregnancy rates) of their ovarian reserve [174].

## 7. Conclusions

In summary, the approach to ovarian cysts and masses in pediatric populations emphasizes careful monitoring and a conservative approach due to the differences in presentation and management compared to adults, reserving intervention for symptomatic cases or those with concerning features. Each case should be evaluated individually, tailoring the management strategy to the patient’s specific circumstances. Multidisciplinary teams, consisting of pediatric gynecologists, pediatric surgeons, and pediatric oncologists (if malignancy is suspected) in specialized centers should treat children and adolescents with an ovarian mass, which can provide optimal physical and psychological support, avoiding unnecessary oophorectomies and ensuring the preservation of ovarian function to maintain hormonal balance, fertility, and overall health.

## Figures and Tables

**Table 1:** Differential diagnosis of adnexal masses in pediatric patients.

Gynecologic	Nongynecologic
Functional cysts Corpus luteum cysts Tubal cysts Paratubal cysts Paraovarian cysts Polycystic ovaries Müllerian anomalies Tubo-ovarian abscess Pelvic inflammatory disease Endometrioma Hydrosalpinx Pyosalpinx Dilated (obstructed) vagina Ectopic pregnancy	Ureterocele Urachal cysts Megaureter Ureteral duplication Cystic renal disease Pelvic kidney Wilms’ tumor Hydronephrosis Neuroblastoma Lymphoma Mesenteric or omental cysts Meckel’s diverticulum Volvulus Intestinal duplication Choledochal, splenic, or pancreatic cysts Lymphangioma Lymphadenopathy Peritoneal inclusion cysts Appendicitis Appendiceal or pelvic abscess Constipation Ascites related to various conditions

## Data Availability

Not applicable.

## Abbreviations

The following abbreviations are used in this manuscript:

OCPs	Oral contraceptive pills
YST	Yolk sac tumor
GCT	Germ cell tumor
IOTA	International Ovarian Tumor Analysis
O-RADS	Ovarian-Adnexal Reporting and Data System
CT	Computed tomography
MRI	Magnetic resonance imaging
AFP	Alpha-fetoprotein
LDH	Lactate dehydrogenase
β-hCG	Beta subunit of human chorionic gonadotropin
CA-125	Cancer antigen 125
HE4	Human epididymis protein 4
CEA	Carcinoembryonic antigen
ROMA	Risk of Ovarian Malignancy Algorithm
GOG	Gynecologic Oncology Group
COG	Children’s Oncology Group
FIGO	International Federation of Gynecology and Obstetrics
MIS	Minimally invasive surgery
